# Supplementary material for: Booster Vaccination Decreases 28-Day All-Cause Mortality of the Elderly Hospitalized Due to SARS-CoV-2 Delta Variant
Source: Vaccines (Basel). 2022 Jun 21;10(7):986. doi: 10.3390/vaccines10070986 (PMC9321254; doi:10.3390/vaccines10070986)
Supplement: Supplementary file 1 [file vaccines-10-00986-s001.zip › vaccines-1727683-supplementary.pdf]

vaccines

MDPI

Article

Supplementary material

Table S1. The calculated odds ratios (OR) in the multivariate models predicting hospitalization and 28-day all-cause mortality in the infected population

	OR	95% confidence interval
Predicting hospitalization		
Male sex	1.545*	[1.484 - 1.609]
Age (1 year)	1.063*	[1.06 - 1.065]
Heart failure	1.383*	[1.278 - 1.497]
COPD	1.498*	[1.394 - 1.61]
Diabetes type 2	1.587*	[1.519 - 1.658]
Malignancy	1.183*	[1.118 - 1.251]
Primary vaccination	0.363*	[0.348 - 0.378]
Booster vaccination	0.204*	[0.19 - 0.218]
Predicting 28-day all cause mortality after hospitalization		
Male	1.789*	[1.687 - 1.898]
Age (1 years)	1.080*	[1.076 - 1.084]
Heart failure	1.500*	[1.353 - 1.662]
COPD	1.340*	[1.212 - 1.481]
Diabetes type 2	1.604*	[1.507 - 1.706]
Malignancy	1.164*	[1.074 - 1.262]
Primary vaccination	0.322*	[0.303 - 0.342]
Booster vaccination	0.198*	[0.178 - 0.221]

***p<0.000**

Table S2. The absolute distribution of vaccination variations of the primary and booster vaccinated populations at the beginning of the observation period

13 September, 2021

**3rd vaccine type**	**2nd vaccine type**	**1st vaccine type**					
		Astra	Moderna	Pfizer	Sinopharm	Sputnik V	Janssen*
None	Astra	135132	50	88	194	566	11813
	Moderna	180	97816	128	215	26	
	Pfizer	962	207	655950	1938	312	
	Sinopharm	200	141	496	317968	175	
	Sputnik V	19	32	263	64	152689	
		Astra	Moderna	Pfizer	Sinopharm	Sputnik V	Janssen*
Astra	Astra	0	0	1	0	0	0
	Moderna	0	50	0	0	0	

	Pfizer	1	0	460	0	0	
	Sinopharm	0	0	0	209	0	
	Sputnik V	0	0	0	0	12	
		Astra	Moderna	Pfizer	Sinopharm	Sputnik V	Janssen*
Moderna	Astra	35	0	0	1	1	0
	Moderna	0	1853	7	2	0	
	Pfizer	0	0	652	1	0	
	Sinopharm	2	2	1	4686	3	
	Sputnik V	0	0	0	3	571	
		Astra	Moderna	Pfizer	Sinopharm	Sputnik V	Janssen*
Pfizer	Astra	329	1	1	31	0	8
	Moderna	2	2452	13	12	0	
	Pfizer	4	4	54520	33	7	
	Sinopharm	45	15	34	141299	40	
	Sputnik V	3	2	38	4	13873	
		Astra	Moderna	Pfizer	Sinopharm	Sputnik V	Janssen*
Sinopharm	Astra	7	0	0	0	0	0
	Moderna	0	626	1	0	1	
	Pfizer	0	0	3428	0	0	
	Sinopharm	1	1	1	766	0	
	Sputnik V	0	0	1	0	131	
		Astra	Moderna	Pfizer	Sinopharm	Sputnik V	Janssen*
Sputnik V	Astra	0	0	0	0	0	0
	Moderna	0	12	0	0	0	
	Pfizer	0	0	97	0	0	
	Sinopharm	0	0	0	87	0	
	Sputnik V	0	0	0	0	8	
		Astra	Moderna	Pfizer	Sinopharm	Sputnik V	Janssen*
Janssen	Astra	2	0	0	1	0	0
	Moderna	0	796	2	1	0	
	Pfizer	0	2	3557	1	1	
	Sinopharm	4	3	0	3913	1	
	Sputnik V	0	0	2	0	200	

Table S3. The absolute distribution of vaccination variations of the primary and booster vaccinated populations at the end of the observation period

		31 December, 2021					
**3rd vaccine type**	**2nd vaccine type**	**1st vaccine type**					
		Astra	Moderna	Pfizer	Sinopharm	Sputnik V	Janssen*
None	Astra	32564	18	43	51	124	12443
	Moderna	177	30817	160	388	73	

	Pfizer	1721	621	190953	5703	1122	
	Sinopharm	81	70	299	57525	43	
	Sputnik V	4	9	72	21	31606	
		Astra	Moderna	Pfizer	Sinopharm	Sputnik V	Janssen*
Astra	Astra	461	0	1	1	6	0
	Moderna	1	183	0	1	0	
	Pfizer	1	0	1008	1	1	
	Sinopharm	0	0	0	325	0	
	Sputnik V	0	0	0	0	19	
		Astra	Moderna	Pfizer	Sinopharm	Sputnik V	Janssen*
Moderna	Astra	7655	7	6	9	33	334
	Moderna	63	36759	27	51	9	
	Pfizer	17	14	13096	14	3	
	Sinopharm	13	35	7	19274	8	
	Sputnik V	2	1	6	5	9552	
		Astra	Moderna	Pfizer	Sinopharm	Sputnik V	Janssen*
Pfizer	Astra	92326	27	45	155	395	2913
	Moderna	53	29090	69	58	5	
	Pfizer	571	76	509588	368	170	
	Sinopharm	166	75	300	371395	156	
	Sputnik V	14	22	222	45	122473	
		Astra	Moderna	Pfizer	Sinopharm	Sputnik V	Janssen*
Sinopharm	Astra	720	0	0	3	3	66
	Moderna	2	2516	4	1	1	
	Pfizer	2	1	10615	15	1	
	Sinopharm	7	6	10	7338	0	
	Sputnik V	0	1	5	1	1423	
		Astra	Moderna	Pfizer	Sinopharm	Sputnik V	Janssen*
Sputnik V	Astra	6	0	0	0	0	0
	Moderna	0	23	0	0	0	
	Pfizer	0	0	200	0	0	
	Sinopharm	1	0	0	130	0	
	Sputnik V	0	0	1	0	70	
		Astra	Moderna	Pfizer	Sinopharm	Sputnik V	Janssen*
Janssen	Astra	286	0	1	1	0	481
	Moderna	1	3573	3	4	0	
	Pfizer	2	7	12552	12	4	
	Sinopharm	8	4	3	10174	16	
	Sputnik V	0	1	5	0	1650	
